# QuantumInformation.jl—A Julia package for numerical computation in quantum information theory

**DOI:** 10.1371/journal.pone.0209358

**Published:** 2018-12-26

**Authors:** Piotr Gawron, Dariusz Kurzyk, Łukasz Pawela

**Affiliations:** Institute of Theoretical and Applied Informatics, Polish Academy of Sciences, Bałtycka 5, 44-100 Gliwice, Poland; Durham University, UNITED KINGDOM

## Abstract

Numerical investigations are an important research tool in quantum information theory. There already exists a wide range of computational tools for quantum information theory implemented in various programming languages. However, there is little effort in implementing this kind of tools in the Julia language. Julia is a modern programming language designed for numerical computation with excellent support for vector and matrix algebra, extended type system that allows for implementation of elegant application interfaces and support for parallel and distributed computing. QuantumInformation.jl is a new quantum information theory library implemented in Julia that provides functions for creating and analyzing quantum states, and for creating quantum operations in various representations. An additional feature of the library is a collection of functions for sampling random quantum states and operations such as unitary operations and generic quantum channels.

## Introduction

Numerical investigations are prevalent in quantum information theory. Numerical experiments can be used to find counter examples for theorems, to test hypotheses or to gain insight about quantum objects and operations.

The variety of software that supports investigations in quantum information theory is very large. Yet there are niches that are not well covered. The purpose of QuantumInformation.jl library is to provide functions to create quantum states, manipulate them with quantum channels, calculate functionals on these objects and sample them randomly from various distributions. QuantumInformation.jl package is available on-line at https://github.com/ZKSI/QuantumInformation.jl and stored at Zenodo repository [1]. It is published under GNU General Public License v3.0.

### Related work

A comprehensive collection of software related to quantum mechanics, computation and information can be found at Quantiki [2]—an on-line resource for quantum information research community. There exist several notable libraries aimed at numerical and symbolic computation for quantum information theory. Two Mathemetica libraries—QI [3] and TRQS [4]—were an inspiration for creation of QuantumInformation.jl. Additionally the QUANTUM [5] library was implemented in Mathematica. A library called FEYNMAN implemented in Maple, described in a series of papers [6–10], provides a wide variety of functions. The above-mentioned libraries rely on non-free software and therefore their use can be very limited as use of this software requires acquiring expensive licenses and its source code cannot be studied by researchers. Therefore any results obtained using this software rely on trust to the companies that produced it. Hence non-free software creates barriers for reproducibility of scientific results [11].

A widely celebrated and used framework QuTiP [12, 13] was written in Python. Python posses many scientific computation libraries. It is free software and is widely used for scientific computation. Nevertheless, as a general purpose programming language it has its limits. In Python, implementations of multidimensional arrays and linear algebra routines are provided by NumPy [14] and SciPy [15] respectively. Unfortunately, due to low efficiency of Python, many of the underling functions are implemented in C or Fortran programming languages. Therefore, study and development of these routines is difficult and requires familiarity with these low-level languages.

Julia [16], being a high-level just-in-time compiled language, is very efficient and therefore extremely useful for scientific computing. There are several libraries related to quantum mechanics and quantum information written in Julia. Those are: QuantumInfo.jl [17], Quantum.jl [18] and a collection of packages developed as a part of JuliaQuantum project [19]. Unfortunately these development efforts stalled a couple of years ago. JuliaQuantum project is very ambitious, but its scope seems to be too large to be implemented fully in a relatively short amount of time. The only package whose development was successful is QuantumOptics.jl—a Julia framework for simulating open quantum systems [20]. Yet the applicability scope of this package is different than the one of QuantumInformation.jl.

### Design principles

Our goal while designing QuantumInformation.jl library was to follow the principles presented in the book “Geometry of Quantum States” [21]. We work with column vectors representing kets and row vectors representing bras. We fix our basis to the computational one. Density matrices and quantum channels are represented as two-dimensional arrays in the same fixed basis. This approach allows us to obtain a low level of complexity of our code, high flexibility and excellent computational efficiency. The design choices were highly motivated by the properties of the language in which our library was implemented, namely Julia [22].

Julia is a novel scientific programming language mainly influenced by Python, Matlab and Lisp programming languages. One of the main concepts widely used in Julia is multiple dispatch *i.e.* an ability to dispatch function calls to different methods depending on the types of all function arguments. The multiple dispatch mechanism together with a simple yet flexible type system allows to build clean and easy to use programming interfaces. Julia is just-in-time compiled to machine code using LLVM [23] therefore, despite being a high-level programming language, it can reach computation efficiency similar to C or Fortran. Julia natively supports parallel and distributed computing techniques. Therefore it is easy to write programs for Monte-Carlo sampling in Julia.

In Julia arrays are first class objects [24], and linear algebra operations are integrated into the language standard library. The array system in Julia is designed in a way that minimizes the amount of memory copying operations during transformations of arrays. Julia supports various representations of vectors and matrices. For these reasons a design decision was made not to create library specific types but to rely on built-in standard library abstract array types.

The QuantumInformation.jl library was initially developed in Julia 0.6 but then subsequently it was ported to Julia version 1.0. Part of the functionality of the library, namely the function that calculates the diamond norm of a quantum channel relies on Convex.jl library [25]. Partial traces are implemented using TensorOperations.jl library [26] that provides basic tensor contractions primitives.

### Testing

The QuantumInformation.jl library was tested using standard Julia framework. Tests where performed using three distinct approaches. In case of most of the functions the basic properties, such as e.g. dimensions, norms, hermititicty, positivity, trace are tested, where it was appropriate. Additionally some test cases where manually computed and used to verify the obtained results. In the case of methods generating random objects such as random matrices statistical properties of results are tested. For example in case of random unitary matrices sampling we test phases distribution [27] of obtained matrices in order to ensure that the unitary matrices are drawn according to the Haar measure.

### Organization of the paper

In the section **Linear algebra in Julia**, we describe briefly how the linear algebra routines are implemented in Julia. Next, in the section **States and channels**, we introduce the notions of *quantum states* and *quantum channels* and we discuss how we implement these concepts in Julia. Subsequently, the section **Functionals** focuses on functionals related with quantum information processing, *i.e.* *trace norm, diamond norm, entropy, fidelity* or the *PPT criterion*. Afterward, we show the usage of QuantumInformation.jl for modeling and application of the *quantum measurements*. The section **Random quantum objects** introduces probabilistic measures on quatum states and channels and their implementation in Julia. Additionally, we introduce some common random matrix ensembles. In section **Benchmarks** we provide a comparison, in terms of code clarity and execution speed, of our library with the latest version of QuTiP [12, 13]. Finally, in the section **Conclusions and future work** we present the final remarks and outline possible future work.

## Linear algebra in Julia

A basic construction of vector in Julia creates a full one-index array containing elements of a number type as presented below.

julia> x = [0.0, 1.0im]

2-element Array{Complex{Float64},1}:

 0.0 + 0.0im

 0.0 + 1.0im

A transposition of a column vector returns an object of type LinearAlgebra.Transpose as shown below

julia> xt = transpose(x)

1×2 LinearAlgebra.Transpose{Complex{Float64},Array{Complex{Float64},1}}:

 0.0 + 0.0im 0.0 + 1.0im

While a Hermitian conjugate of the same vector returns a LinearAlgebra.Adjoint parametrized by the type Array:

julia> xc = [0.0, 1.0im]’

1×2 LinearAlgebra.Adjoint{Complex{Float64},Array{Complex{Float64},1}}:

 0.0-0.0im 0.0-1.0im

Values of variables xt and xc are views of the value of variable x. The column and row vectors behave like bras and kets, for example xc*x denotes the inner product of ‘bra’ xc and ‘ket’ x, while x*xc denotes their outer product resulting in a two-index array.

The linear algebra library in Julia provides standard operations on matrices and vectors that are designed to take into account the types of the objects.

## States and channels

In this and the following sections we will denote complex Euclidean spaces ℂ^*d*^ with X, Y, 𝒵 etc. When needed the dimension of a space X will be denoted dim(X). The set of matrices transforming vectors from X to Y will be denoted L(X,Y). For simplicity we will write L(X)≡L(X,X).

### States

By |ψ〉∈X we denote a normed column vector. Notice that any |*ψ*〉 can be expressed as $|\psi \rangle =\sum_{i=1}^{n}\alpha_{i}|i\rangle$, where $\sum_{i=1}^{n}{\left|\alpha_{i}\right|}^{2}=1$ and the set ${\{|i\rangle \}}_{i=1}^{n}$ is the computational basis.

julia> ket(1,2)

2-element Array{Complex{Float64},1}:

 1.0 + 0.0im

 0.0 + 0.0im

julia> (1/sqrt(2)) * (ket(1,2) + ket(2,2))

2-element Array{Complex{Float64},1}:

 0.7071067811865475 + 0.0im

 0.7071067811865475 + 0.0im

According to common academic convention, we count the indices of states starting from one. Following the standard Dirac notation the symbol 〈*ψ*| denotes the row vector dual to |*ψ*〉. Therefore |*ψ*〉 = 〈*ψ*|^†^, where the symbol ^†^ denotes the Hermitian conjugation.

julia> bra(2,3)

1×3 LinearAlgebra.Adjoint{Complex{Float64},Array{Complex{Float64},1}}:

 0.0-0.0im 1.0-0.0im 0.0-0.0im

The inner product of |ϕ〉,ψ〉∈X is denoted by 〈*ψ*|*ϕ*〉 and the norm is defined as $∥\;|\phi \rangle ∥=\sqrt{\langle \phi |\phi \rangle }$.

julia> *ψ* = (1/sqrt(2)) * (ket(1,2) + ket(2,2))

2-element Array{Complex{Float64},1}:

 0.7071067811865475 + 0.0im

 0.7071067811865475 + 0.0im

julia> *ϕ* = (1/2) * ket(1,2) + (sqrt(3)/2) * ket(2,2)

2-element Array{Complex{Float64},1}:

 0.5 + 0.0im

 0.8660254037844386 + 0.0im

julia> *ϕ*’ * *ψ*

 0.9659258262890682 + 0.0im

julia> sqrt(*ϕ*’ * *ϕ*)

 0.9999999999999999 + 0.0im

The form |ψ〉〈ϕ|∈L(X,Y) denotes outer product of |ψ〉∈Y and |ϕ〉∈X.

julia> ketbra(2,3,4)

4×4 Array{Complex{Float64},2}:

 0.0+0.0im 0.0+0.0im 0.0+0.0im 0.0+0.0im

 0.0+0.0im 0.0+0.0im 1.0+0.0im 0.0+0.0im

 0.0+0.0im 0.0+0.0im 0.0+0.0im 0.0+0.0im

 0.0+0.0im 0.0+0.0im 0.0+0.0im 0.0+0.0im

Specifically, |*ψ*〉〈*ψ*| is a rank-one projection operator called a *pure state*. Generally, any *quantum state* *ρ* can be expressed as $\rho =\sum_{i=1}^{n}q_{i}|\psi_{i}\rangle \langle \psi_{i}|$, where $\sum_{i=1}^{n}q_{i}=1$ and |*ψ*_*i*_〉〈*ψ*_*i*_| are rank-one projectors. Notice that *ρ* is a trace-one positive semi-definite linear operator *i.e.*: *ρ* = *ρ*^†^, *ρ* ≥ 0 and tr*ρ* = 1.

julia> proj(ψ)

2×2 Array{Complex{Float64},2:

 0.5+0.0im 0.5+0.0im

 0.5+0.0im 0.5+0.0im

For convenience, the QuantumInformation.jl library provides the implementations of maximally mixed, maximally entangled and Werner states.

julia> max_entangled(4)

4-element reshape(::Diagonal{Complex{Float64},Array{Complex{Float64},1}},4)

with eltype {Complex{Float64}:

 0.7071067811865475 + 0.0im

 0.0 + 0.0im

 0.0 + 0.0im

 0.7071067811865475 + 0.0im

julia> max_mixed(4)

4×4 Array{Float64,2}:

0.25 0.0 0.0 0.0

0.0 0.25 0.0 0.0

0.0 0.0 0.25 0.0

0.0 0.0 0.0 0.25

julia> werner_state(4, 0.4)

4×4 Array{Complex{Float64},2}:

 0.35+0.0im 0.0+0.0im 0.0+0.0im 0.2+0.0im

 0.0+0.0im 0.15+0.0im 0.0+0.0im 0.0+0.0im

 0.0+0.0im 0.0+0.0im 0.15+0.0im 0.0+0.0im

 0.2+0.0im 0.0+0.0im 0.0+0.0im 0.35+0.0im

### Non-standard matrix transformations

We will now introduce reshaping operators, which map matrices to vectors and vice versa. We start with the mapping res:L(X,Y)→Y⊗X, which transforms the matrix *ρ* into a vector row by row. More precisely, for dyadic operators |*ψ*〉〈*ϕ*|, where |ψ〉∈Y, |ϕ〉∈X the operation *res* is defined as $\text{res}(|\psi \rangle \langle \phi |)=\left|\psi \rangle \right|\bar{\phi }\rangle$ and can be uniquely extend to the whole space L(X,Y) by linearity.

julia> res(ketbra(1,2,2))

4-element reshape(::LinearAlgebra.Transpose{Complex{Float64},Array{Complex{Float64},2}}, 4)

with eltype {Complex{Float64}:

 0.0 + 0.0im

 1.0 + 0.0im

 0.0 + 0.0im

 0.0 + 0.0im

The inverse operation to res is unres:Y⊗X→L(X,Y), which transforms the vector into a matrix. It is defined as the unique linear mapping satisfying *ρ* = unres(res(*ρ*)).

julia> unres(res(ketbra(1,2,2)))

2×2 LinearAlgebra.Transpose{Complex{Float64},Base.ReshapedArray{Complex{Float64},2,LinearAlgebra.

Transpose{Complex{Float64},Array{Complex{Float64},2}},

Tuple{Base.MultiplicativeInverses.SignedMultiplicativeInverse{Int64}}}}:

 0.0+0.0im 1.0+0.0im

 0.0+0.0im 0.0+0.0im

Let us recall that trace is a mapping Tr:L(X)→C, given by $\text{Tr}:\rho \mapsto \sum_{i=1}^{\text{dim}(X)}\langle e_{i}\left|\rho \right|e_{i}\rangle$, where {|*e*_*i*_〉} is an orthonormal basis of X. According to this, *partial trace* is a mapping ${\text{Tr}}_{X}:\text{L}\left(X⊗Y\right)\to \text{L}\left(Y\right)$ such that ${\text{Tr}}_{X}:\rho_{A}⊗\rho_{B}\mapsto \rho_{B}\text{Tr}\left(\rho_{A}\right)$, where $\rho_{A}\in \text{L}\left(X\right)$, $\rho_{B}\in \text{L}\left(Y\right)$. As this is a linear map, it may be uniquely extended to the case of operators which are not in a tensor product form.

julia> *ρ* = [0.25 0.25im; -0.25im 0.75]

2×2 Array{Complex{Float64},2}:

 0.25+0.0im 0.0+0.25im

 -0.0-0.25im 0.75+0.0im

julia> *σ* = [0.4 0.1im; -0.1im 0.6]

2×2 Array{Complex{Float64},2:

 0.4+0.0im 0.0+0.1im

 -0.0-0.1im 0.6+0.0im

julia> ptrace(*ρ* ⊗ *σ*, [2, 2], [2])

2×2 Array{Complex{Float64},2}:

 0.25+0.0im 0.0+0.25im

 0.0-0.25im 0.75+0.0im

Matrix transposition is a mapping ${}^{T}:\text{L}\left(X,Y\right)\to \text{L}\left(Y,X\right)$ such that (*ρ*^*T*^)_*ij*_ = *ρ*_*ji*_, where *ρ*_*ij*_ is a *i*-th row, *j*-th column element of matrix *ρ*. Following this, we may introduce *partial transposition* ${}^{\Gamma_{B}}:\text{L}\left(X_{A}⊗X_{B},Y_{A}⊗Y_{B}\right)\to \text{L}\left(X_{A}⊗Y_{B},Y_{A}⊗X_{B}\right)$, which for a product state *ρ*_*A*_ ⊗ *ρ*_*B*_ is given by ${}^{\Gamma_{B}}:\rho_{A}⊗\rho_{B}\mapsto \rho_{A}⊗\rho_{B}^{T}$. The definition of partial transposition can be uniquely extended for all operators from linearity.

julia> ptranspose(*ρ* ⊗ *σ*, [2, 2], [1])

4×4 Array{Complex{Float64},2}:

 0.1+0.0im 0.0+0.025im 0.0-0.1im 0.025-0.0im

 0.0-0.025im 0.15+0.0im -0.025+0.0im 0.0-0.15im

 0.0+0.1im -0.025+0.0im 0.3+0.0im 0.0+0.075im

 0.025-0.0im 0.0+0.15im 0.0-0.075im 0.45+0.0im

For given multiindexed matrix *ρ*_(*m*,*μ*),(*n*,*ν*)_ = 〈*mμ*|*ρ*|*nν*〉, the reshuffle operation is defined as $\rho_{\left(m,\mu ),(n,\nu \right)}^{R}=\rho_{\left(m,n),(\mu ,\nu \right)}$.

julia> reshuffle(*ρ* ⊗ σ)

4×4 Array{Complex{Float64},2}:

 0.1+0.0im 0.0+0.025im 0.0-0.025im 0.15+0.0im

 0.0+0.1im -0.025+0.0im 0.025-0.0im 0.0+0.15im

 0.0-0.1im 0.025-0.0im -0.025+0.0im 0.0-0.15im

 0.3+0.0im 0.0+0.075im 0.0-0.075im 0.45+0.0im

### Channels

Physical transformations of quantum states into quantum states are called quantum channels *i.e.* linear Completely Positive Trace Preserving (CP-TP) transformations. Probabilistic transformations of quantum states are called quantum operations and mathematically they are defined as linear Completely Positive Trace Non-increasing (CP-TNI) maps. For the sake of simplicity we will refer to both CP-TP and CP-TNI maps as quantum channels when it will not cause confusion.

There exists various representations of quantum channels such as:

- Kraus operators,
- natural representation, also called superoperator representation,
- Stinespring representation,
- Choi-Jamiołkowski matrices, sometimes called dynamical matrices.

Formally, properties of quantum channels can be stated as follows [28]. First, we introduce the notion of *superoperator* as a linear mapping acting on linear operators L(X) and transforming them into operators acting on L(Y). The set of all such mapping will be denoted by T(X,Y) and T(X)≡T(X,X). In mathematical terms, a quantum channel is a superoperator Φ:L(X)→L(Y) that is

- *trace-preserving* (∀ρ∈L(X)Tr(Φ(ρ))=Tr(ρ)) and
- *completely positive* ($\forall 𝒵\forall \rho \in \text{L}\left(X⊗𝒵\right),\rho \geq 0,\Phi ⊗I_{\text{L}(𝒵)}\left(\rho \right)\geq 0$).

The product of superoperators $\Phi_{1}\in T\left(X_{1},Y_{1}\right)$, $\Phi_{2}\in T\left(X_{2},Y_{2}\right)$ is a mapping $\Phi_{1}⊗\Phi_{2}\in T\left(X_{1}⊗X_{2},Y_{1}⊗Y_{2}\right)$ that satisfies (Φ_1_ ⊗ Φ_2_)(*ρ*_1_ ⊗ *ρ*_2_) = Φ_1_(*ρ*_1_) ⊗ Φ_2_(*ρ*_2_). For the operators that are not in a tensor product form this notion can be uniquely extended from linearity.

According to Kraus’ theorem, any completely positive trace-preserving (CP-TP) map Φ can always be written as $\Phi \left(\rho \right)=\sum_{i=1}^{r}K_{i}\rho K_{i}^{†}$ for some set of operators ${\left\{K_{i}\right\}}_{i=1}^{r}$ satisfying $\sum_{i=1}^{r}K_{i}^{†}K_{i}=I_{X}$, where *r* is the rank of superoperator Φ.

Another way to represent the quantum channel is based on Choi-Jamiołkowski isomorphism. Consider mapping J:T(X,Y)→L(Y⊗X) such that $J\left(\Phi \right)=\left(\Phi ⊗I_{\text{L}(X)}\right)\left(\text{res}\left(I_{X}\right)\text{res}{\left(I_{X}\right)}^{†}\right)$. Equivalently $J\left(\Phi \right)=\sum_{i,j=1}^{\text{dim}(X)}\Phi (|i\rangle \langle j|)⊗\left|i\rangle \langle j\right|$. The action of a superoperator in the Choi representation is given by $\Phi \left(\rho \right)={\text{Tr}}_{X}\left(J\left(\Phi \right)\left(I_{Y}⊗\rho^{T}\right)\right)$.

The natural representation of a quantum channel T(X,Y) is a mapping res(*ρ*) ↦ res(Φ(*ρ*)). It is represented by a matrix K(Φ)∈L(X⊗X,Y⊗Y) for which the following holds

$$\begin{array}{c} K(\Phi )\text{res}(\rho )=\text{res}(\Phi (\rho )), \end{array}$$ (1)

for all ρ∈L(X).

Let X,Y and 𝒵 be a complex Euclidean spaces. The action of the Stinespring representation of a quantum channel Φ∈T(X,Y) on a state ρ∈L(X) is given by

$$\begin{array}{c} \Phi \left(\rho \right)={\text{Tr}}_{𝒵}\left(A\rho A^{†}\right), \end{array}$$ (2)

where A∈L(X,Y⊗𝒵).

We now briefly describe the relationships among channel representations [28]. Let Φ∈T(X,Y) be a quantum channel which can be written in the Kraus representation as

$$\begin{array}{c} \Phi \left(\rho \right)=\sum_{i=1}^{r}K_{i}\rho K_{i}^{†}, \end{array}$$ (3)

where ${\left\{K_{i}\right\}}_{i=1}^{r}$ are Kraus operators satisfying $\sum_{i=1}^{r}K_{i}^{†}K_{i}=I_{X}$. According to this assumption, Φ can be represented in

- Choi representation as
  $$\begin{array}{c} J\left(\Phi \right)=\sum_{i=1}^{r}\text{res}\left(K_{i}\right)\text{res}\left(K_{i}^{†}\right), \end{array}$$ (4)
- natural representation as
  $$\begin{array}{c} K\left(\Phi \right)=\sum_{i=1}^{r}K_{i}⊗K_{i}^{*}, \end{array}$$ (5)
- Stinespring representation as
  $$\begin{array}{c} \Phi \left(\rho \right)={\text{tr}}_{𝒵}\left(A\rho A^{†}\right), \end{array}$$ (6)
  where $A=\sum_{i=1}^{r}K_{i}⊗e_{i}\rangle$ and $𝒵={\mathbb{C}}^{r}$.

In QuantumInformation.jl states and channels are always represented in the computational basis therefore channels are stored in the memory as either vectors of matrices in case of Kraus operators or matrices in other cases. In QuantumInformation.jl quantum channels are represented by a set of types deriving from an abstract type AbstractQuantumOperation{T} where type parameter T should inherit from AbstractMatrix{<:Number}. Every type inheriting from AbstractQuantumOperation{T} should contain fields idim and odim representing the dimension of input and output space of the quantum channel.

Two special types of channels are implemented: UnitaryChannel and IdentityChannel that can transform ket vectors into ket vectors.

#### Constructors

Channel objects can be constructed from matrices that represent them, as shown in the following listing

julia> *γ* = 0.4

 0.4

julia> K0 = Matrix([1 0; 0 sqrt(1-*γ*)])

2×2 Array{Float64,2}:

 1.0 0.0

 0.0 0.774597

julia> K1 = Matrix([0 sqrt(*γ*); 0 0])

2×2 Array{Float64,2}:

 0.0 0.632456

 0.0 0.0

julia> Φ = KrausOperators([K0,K1])

KrausOperators{Array{Float64,2}}

dimensions: (2, 2)

 [1.0 0.0; 0.0 0.774597]

 [0.0 0.632456; 0.0 0.0]

julia> iscptp(Φ)

 true

There are no checks whether a matrix represents a valid CP-TP or CP-TNI map, because this kind of verification is costly and requires potentially expensive numerical computation. Function such as iscptp(), and iscptni() are provided to test properties of supposed quantum channel or quantum operation.

#### Conversion

Conversions between all quantum channel types, *i.e.* these that derive from AbstractQuantumOperation{T} are implemented. The users are not limited by any single channel representation and can transform between representations they find the most efficient or suitable for their purpose.

julia> Ψ1 = convert(SuperOperator{Matrix{ComplexF64}}, Φ)

SuperOperator{Array{Complex{Float64},2}}

dimensions: (2, 2)

Complex{Float64}

 [1.0+0.0im 0.0+0.0im 0.0+0.0im 0.4+0.0im;

 0.0+0.0im 0.774597+0.0im 0.0+0.0im 0.0+0.0im;

 0.0+0.0im 0.0+0.0im 0.774597+0.0im 0.0+0.0im;

 0.0+0.0im 0.0+0.0im 0.0+0.0im 0.6+0.0im]

julia> Ψ2 = convert(DynamicalMatrix{Matrix{Float64}}, Φ)

DynamicalMatrix{Array{Float64,2}}

dimensions: (2, 2)

 [1.0 0.0 0.0 0.774597;

 0.0 0.4 0.0 0.0;

 0.0 0.0 0.0 0.0;

 0.774597 0.00.0 0.6]

julia> Ψ3 = convert(Stinespring{Matrix{Float64}}, Φ)

Stinespring{Array{Float64,2}}

dimensions: (2, 2)

 [0.0 0.0;

 -1.82501e-8 0.0;

 …;

 0.0 0.0;

 0.0 -0.774597]

#### Application

Channels can act on pure and mixed states represented by vectors and matrices respectively. Channels are callable and therefore mimic application of a function on a quantum state.

julia> *ρ*1 = *ψ* * *ψ*’

2×2 Array{Complex{Float64},2}:

 0.5+0.0im 0.5+0.0im

 0.5+0.0im 0.5+0.0im

julia> Φ(*ρ*1)

2×2 Array{Complex{Float64},2}:

 0.7+0.0im 0.387298+0.0im

 0.387298+0.0im 0.3+0.0im

julia> Ψ1(*ρ*1)

2×2 Array{Complex{Float64},2}:

 0.7+0.0im 0.387298+0.0im

 0.387298+0.0im 0.3+0.0im

julia> Φ(*ψ*)

2×2 Array{Complex{Float64},2}:

 0.7+0.0im 0.387298+0.0im

 0.387298+0.0im 0.3+0.0im

#### Composition

Channels can be composed in parallel or in sequence. Composition in parallel is done using kron() function or the overloaded ⊗ operator. Composition in sequence can be done in two ways either by using Julia built-in function composition operator (*f* ∘ *g*)(⋅) = *f*(*g*)(⋅) or by using multiplication of objects inheriting from AbstractQuantumOperation{T} abstract type.

julia> *ρ*2 = *ϕ* * *ϕ*’

2×2 Array{Complex{Float64},2}:

 0.25+0.0im   0.433013+0.0im

 0.433013+0.0im 0.75+0.0im

julia> (Φ ⊗ Φ)(*ρ*1 ⊗ *ρ*2)

4×4 Array{Complex{Float64},2}:

 0.385+0.0im   0.234787+0.0im 0.213014+0.0im 0.129904+0.0im

 0.234787+0.0im 0.315+0.0im   0.129904+0.0im 0.174284+0.0im

 0.213014+0.0im 0.129904+0.0im 0.165+0.0im   0.100623+0.0im

 0.129904+0.0im 0.174284+0.0im 0.100623+0.0im 0.135+0.0im

julia> (Ψ1 ∘ Ψ2)(*ρ*1)

2×2 Transpose{Complex{Float64},Array{Complex{Float64},2}:

0.82+0.0im 0.3+0.0im

0.3+0.0im 0.18+0.0im

## Functionals

### Trace norm and distance

Let ρ,σ∈L(X). The *trace norm* is defined as ${∥\rho ∥}_{1}=\text{Tr}\sqrt{\rho \rho^{†}}$ and the trace distance is defined as $D_{1}\left(\rho ,\sigma \right)=\frac{1}{2}{∥\rho -\sigma ∥}_{1}$.

julia> *ψ* = (1/sqrt(2)) * (ket(1,2) + ket(2,2))

2-element Array{Complex{Float64},1}:

 0.7071067811865475 + 0.0im

 0.7071067811865475 + 0.0im

*ϕ* = (1/2) * ket(1,2) + (sqrt(3)/2) * ket(2,2)

2-element Array{Complex{Float64},1}:

 0.5 + 0.0im

 0.8660254037844386 + 0.0im

julia> *ρ* = proj(*ψ*)

2×2 Array{Complex{Float64},2}:

 0.5+0.0im 0.5+0.0im

 0.5+0.0im 0.5+0.0im

julia> *σ* = proj(*ϕ*)

2×2 Array{Complex{Float64},2}:

 0.25+0.0im 0.433013+0.0im

 0.433013+0.0im 0.75+0.0im

julia> norm_trace(*ρ*)

 1.0

julia> trace_distance(*ρ*, *σ*)

 0.2588190451025207 + 0.0im

### Hilbert–Schmidt norm and distance

The *Hilbert–Schmidt* norm and distance defined by ${∥\rho ∥}_{HS}=\sqrt{\text{Tr}\rho^{†}\rho }$ and $D_{HS}\left(\rho ,\sigma \right)=\frac{1}{2}{∥\rho -\sigma ∥}_{HS}$, respectively, can be used as follows

julia> norm_hs(*ρ*)

 0.9999999999999998

julia> hs_distance(*ρ*, *σ*)

 0.36602540378443854

### Fidelity and superfidelity

*Fidelity* is a measure of distance of quantum states. It is an example of a distance measure which is not a metric on the space of quantum states. The fidelity of two quantum states ρ,σ∈L(X) is given by $F\left(\rho ,\sigma \right)=∥\sqrt{\rho }\sqrt{\sigma }{∥}_{1}$

julia> fidelity_sqrt(*ρ*, *σ*)

 0.9659258262890682

julia> fidelity(*ρ*, *σ*)

 0.9330127018922192

julia> fidelity(*ψ*, *σ*)

 0.9330127018922191

julia> fidelity(*ρ*, *ϕ*)

 0.9330127018922191

julia> fidelity(*ψ*, *ϕ*)

 0.9330127018922192

*Superfidelity* is an upper bound on the fidelity of two quantum states It is defined by $G\left(\rho ,\sigma \right)=\text{Tr}\rho \sigma +\sqrt{1-\text{Tr}\rho^{2}}\sqrt{1-\text{Tr}\sigma^{2}}$.

julia> superfidelity(*ρ*, *σ*)

 0.9330127018922193

### Diamond norm

In order to introduce the *diamond norm*, we first introduce the notion of the induced trace norm. Given Φ∈T(X,Y) we define its induced trace norm as ${∥\Phi ∥}_{1}=\text{max}\{{∥\Phi \left(X\right)∥}_{1}:X\in L\left(X\right),{∥X∥}_{1}\leq 1\}$. The diamond norm of Φ is defined as ${∥\Phi ∥}_{◇}={∥\Phi ⊗I_{\text{L}(Y)}∥}_{1}$. One important property of the diamond norm is that for Hermiticity-preserving Φ∈T(X,Y) we obtain $∥\Phi {∥}_{◇}=\text{max}\left\{∥\left(\Phi ⊗I_{\text{L}\left(Y\right)}\right)\left(\left|\psi \rangle \langle \psi \right|\right){∥}_{1}:|\psi \rangle \in X⊗Y,\langle \psi |\psi \rangle =1\right\}$.

julia> K0 = Matrix([1 0; 0 sqrt(1-*γ*)])

2×2 Array{Float64,2}:

 1.0 0.0

 0.0 0.774597

julia> K1 = Matrix([0 sqrt(*γ*); 0 0])

2×2 Array{Float64,2}:

 0.0 0.632456

 0.0 0.0

julia> Φ = KrausOperators([K0,K1])

KrausOperators{Array{Float64,2}}

dimensions: (2, 2)

 [1.0 0.0; 0.0 0.774597]

 [0.0 0.632456; 0.0 0.0]

julia> L0 = Matrix([1 0; 0 sqrt(1-*γ*)])

2×2 Array{Float64,2}:

 1.0 0.0

 0.0 0.774597

julia> L1 = Matrix([0 0; 0 sqrt(*γ*)])

2×2 Array{Float64,2}:

 0.0 0.0

 0.0 0.632456

julia> Ψ = KrausOperators([K0,K1])

KrausOperators{Array{Float64,2}}

dimensions: (2, 2)

 [1.0 0.0; 0.0 0.774597]

 [0.0 0.632456; 0.0 0.0]

julia> norm_diamond(Φ)

 1.0000000077706912

julia> diamond_distance(Φ, Ψ)

 -5.258429449675825e-7

Diamond norm and diamond distance are implemented using the Convex.jl Julia package [25].

### Shannon entropy and von Neumann entropy

*Shannon entropy* is defined for a probability vector *p* as $H\left(p\right)=-\sum_{i=1}^{n}p_{i}\log_{2}p_{i}$. We also provide an implementation for the point Shannon entropy. It is defined as *h*(*a*) = −*a* log *a* − (1 − *a*) log(1 − *a*).

julia> p = [0.3, 0.2, 0.5]

3-element Array{Float64,1}:

 0.3

 0.2

 0.5

julia> shannon_entropy(p)

 1.0296530140645737

julia> shannon_entropy(0.5)

 0.6931471805599453

For a quantum system described by a state *ρ*, the *von Neumann entropy* is *S*(*ρ*) = −tr*ρ* log *ρ*. Let λ_*i*_, 0 ≤ *i* < *n* be the eigenvalues of *ρ*, then *S*(*ρ*) can be written as $S\left(\rho \right)=-\sum_{i=1}^{n}\lambda_{i}\log\lambda_{i}$.

julia> *ρ* = [0.25 0.25im; -0.25im 0.75]

2×2 Array{Complex{Float64},2}:

 0.25+0.0im 0.0+0.25im

 1-0.0-0.25im 0.75+0.0im

julia> *σ* = [0.4 0.1im; -0.1im 0.6]

2×2 Array{Complex{Float64},2}:

 0.4+0.0im 0.0+0.1im

 -0.0-0.1im 0.6+0.0im

julia> vonneumann_entropy(0.4 * *ρ* + 0.6 * *σ*)

 0.5869295208554555

### Distinguishability between two quantum states

One of the measure of distinguishability between two quantum states is the *quantum relative entropy*, called also Kullback–Leibler divergence, defined as *S*(*ρ*‖*σ*) = −Tr*ρ* log *σ* + Tr*ρ* log *ρ*

julia> relative_entropy(*ρ*, *σ*)

 0.11273751829075163

julia> kl_divergence(*ρ*, *σ*)

 0.11273751829075163

Another type of measure of distinguishability between two quantum state is *quantum Jensen–Shannon divergence* given by $QJS\left(\rho ,\sigma \right)=S(\frac{1}{2}\rho +\frac{1}{2}\sigma )-(\frac{1}{2}S\left(\rho \right)+\frac{1}{2}S\left(\sigma \right))$.

julia> js_divergence(*ρ*, *σ*)

 0.1252860912303596

*The Bures distance* defines an infinitesimal distance between quantum states, and it is defined as $D_{B}=\sqrt{2(1-\sqrt{F(\rho ,\sigma )})}$. The value related with Bures distance is the Bures angle $D_{A}\left(\rho ,\sigma \right)=\arccos\left(\sqrt{F(\rho ,\sigma )}\right)$

julia> bures_distance(*ρ*, *σ*)

 0.24867555729886728

julia> bures_angle(*ρ*, *σ*)

 0.2493208055929498

### Quantum entanglement

One of the entanglement measures is *negativity* defined as $N\left(\rho \right)=\frac{∥\rho^{T_{A}}{∥}_{1}-1}{2}$.

julia> negativity(*ρ* ⊗ *σ*, [2, 2], 2)

-0.0

julia> negativity(proj((1/sqrt(2)*(ket(1,2) ⊗ ket(1,2)-ket(2,2) ⊗ ket(2,2)))), [2, 2], 2)

 0.4999999999999999

julia> log negativity(*ρ* ⊗ *σ*, [2, 2], 2)

-1.1102230246251565e-16

*Positive partial transpose* (the Peres–Horodecki criterion) is a necessary condition of separability of the joint state *ρ*_*AB*_. According PPT criterion, if $\rho^{T_{B}}$ has non negative eigenvalues, then *ρ*_*AB*_ is separable.

julia> ppt(*ρ* ⊗ *σ*, [2, 2], 2)

 0.052512626584708365

julia> ppt(proj((1/sqrt(2)*(ket(1,2) ⊗ ket(1,2)-ket(2,2) ⊗ ket(2,2)))), [2, 2], 2)

-0.4999999999999999

Another way to quantification of quantum entanglement is *Concurrence* [29]. Concurrence of quantum state *ρ* is a strong separability criterion. For two-qubit systems it is defined as *C*(*ρ*) = max(0, λ_1_ − λ_2_ − λ_3_ − λ_4_), where λ_*i*_ are decreasing eigenvalues of $\sqrt{\sqrt{\rho }\tilde{\rho }\sqrt{\rho }}$ with $\tilde{\rho }=\left(\sigma_{y}⊗\sigma_{y}\right)\rho^{*}\left(\sigma_{y}⊗\sigma_{y}\right)$. If *C*(*ρ*) = 0, then *ρ* is separable.

julia> *ρ* = [0.25 0.1im; -0.1im 0.75]

2×2 Array{Complex{Float64},2}:

 0.25+0.0im 0.0+0.1im

-0.0-0.1im 0.75+0.0im

julia> *σ* = [0.4 0.1im; -0.1im 0.6]

2×2 Array{Complex{Float64},2}:

 0.4+0.0im 0.0+0.1im

-0.0-0.1im 0.6+0.0im

julia> concurrence(*ρ* ⊗ *σ*)

 0.0

julia> concurrence(proj(max_entangled(4)))

 0.9999999999999998

## Measurements

Measurements are modeled in two ways:

- as Positive Operator Valued Measures (POVMs),
- measurements with post-selection.

In both cases a measurement is treated as a special case of a quantum channel (operation).

### Positive Operator Valued Measure measurement

A POVM measurement is defined as follows. Let μ:Γ→P(X) be a mapping from a finite alphabet of measurement outcomes to the set of linear positive operators. If $\sum_{\xi \in \Gamma }\mu \left(\xi \right)=I_{X}$ then *μ* is a POVM measurement. The set of positive semi-definite linear operators is defined as P(X)={X∈L(X):〈ψ|X|ψ〉≥0forall|ψ〉∈X}. POVM measurement models the situation where a quantum object is destroyed during the measurement process and quantum state after the measurement does not exists.

We model POVM measurement as a channel θ:L(X)→L(Y), where ${Y=\text{span}\{\xi \rangle \}}_{\xi \in \Gamma }$ such that *θ*(*ρ*) = ∑_*ξ*∈Γ_ tr(*ρ μ*(*ξ*))|*ξ*〉〈*ξ*|. This channel transforms the measured quantum state into a classical state (diagonal matrix) containing probabilities of measuring given outcomes. Note that in QuantumInformation.jl Γ = {1, 2, …, |Γ|} and POVM measurements are represented by the type

POVMMeasurement{T} <: AbstractQuantumOperation{T} where

T<:AbstractMatrix{<:Number}

Predicate function ispovm() verifies whether a list of matrices is a proper POVM.

julia> *ρ* = proj(1.0/sqrt(2)*(ket(1,3)+ket(3,3)))

3×3 Array{Complex{Float64},2}:

 0.5+0.0im 0.0+0.0im 0.5+0.0im

 0.0+0.0im 0.0+0.0im 0.0+0.0im

 0.5+0.0im 0.0+0.0im 0.5+0.0im

julia> E0 = proj(ket(1,3))

3×3 Array{Complex{Float64},2}:

 1.0+0.0im 0.0+0.0im 0.0+0.0im

 0.0+0.0im 0.0+0.0im 0.0+0.0im

 0.0+0.0im 0.0+0.0im 0.0+0.0im

julia> E1 = proj(ket(2,3))+proj(ket(3,3))

3×3 Array{Complex{Float64},2}:

 0.0+0.0im 0.0+0.0im 0.0+0.0im

 0.0+0.0im 1.0+0.0im 0.0+0.0im

 0.0+0.0im 0.0+0.0im 1.0+0.0im

julia> M = POVMMeasurement([E0,E1])

POVMMeasurement{Array{Complex{Float64},2}}

dimensions: (3, 2)

Complex{Float64}

 [1.0+0.0im 0.0+0.0im 0.0+0.0im;

 0.0+0.0im 0.0+0.0im 0.0+0.0im;

 0.0+0.0im 0.0+0.0im 0.0+0.0im]

Complex{Float64}

 [0.0+0.0im 0.0+0.0im 0.0+0.0im;

 0.0+0.0im 1.0+0.0im 0.0+0.0im;

 0.0+0.0im 0.0+0.0im 1.0+0.0im]

julia> ispovm(M)

 true

julia> M(*ρ*)

2×2 LinearAlgebra.Diagonal{Float64,Array{Float64,1}}:

 0.5 .

 . 0.5

### Measurement with post-selection

When a quantum system after being measured is not destroyed one can be interested in its state after the measurement. This state depends on the measurement outcome. In this case the measurement process is defined in the following way.

Let μ:Γ→L(X,Y) be a mapping from a finite set of measurement outcomes to set of linear operators called effects. If $\sum_{\xi \in \Gamma }\mu {\left(\xi \right)}^{†}\mu \left(\xi \right)=I_{X}$ then *μ* is a quantum measurement. Given outcome *ξ* was obtained, the state before the measurement, *ρ*, is transformed into sub-normalized quantum state *ρ*_*ξ*_ = *μ*(*ξ*)*ρμ*(*ξ*)^†^. The outcome *ξ* will be obtained with probability *tr*(*ρ*_*ξ*_).

julia> PM = PostSelectionMeasurement(E1)

PostSelectionMeasurement{Array{Complex{Float64},2}}

dimensions: (3, 3)

Complex{Float64}

 [0.0+0.0im 0.0+0.0im 0.0+0.0im;

 0.0+0.0im 1.0+0.0im 0.0+0.0im;

 0.0+0.0im 0.0+0.0im 1.0+0.0im]

julia> iseffect(PM)

 true

julia> PM(*ρ*)

3×3 Array{Complex{Float64},2}:

 0.0+0.0im 0.0+0.0im 0.0+0.0im

 0.0+0.0im 0.0+0.0im 0.0+0.0im

 0.0+0.0im 0.0+0.0im 0.5+0.0im

In QuantumInformation.jl this kind of measurement is modeled as CP-TNI map with a single Kraus operator *μ*(*ξ*) and represented as

PostSelectionMeasurement{T} <: AbstractQuantumOperation{T} where

T<:AbstractMatrix{<:Number}

Measurement types can be composed and converted to Kraus operators, superoperators, Stinespring representation operators, and dynamical matrices.

julia> *α* = 0.3

 0.3

julia> K0 = ComplexF64[0 0 sqrt(*α*); 0 1 0; 0 0 0]

3×3 Array{Complex{Float64},2}:

 0.0+0.0im 0.0+0.0im 0.547723+0.0im

 0.0+0.0im 1.0+0.0im 0.0+0.0im

 0.0+0.0im 0.0+0.0im 0.0+0.0im

julia> K1 = ComplexF64[1 0 0; 0 0 0; 0 0 sqrt(1 − α)]

3×3 Array{Complex{Float64},2}:

 1.0+0.0im 0.0+0.0im 0.0+0.0im

 0.0+0.0im 0.0+0.0im 0.0+0.0im

 0.0+0.0im 0.0+0.0im 0.83666+0.0im

julia> Φ = KrausOperators([K0,K1])

KrausOperators{Array{Complex{Float64},2}}

dimensions: (3, 3)

Complex{Float64}

 [0.0+0.0im 0.0+0.0im 0.547723+0.0im;

 0.0+0.0im 1.0+0.0im 0.0+0.0im;

 0.0+0.0im 0.0+0.0im 0.0+0.0im]

Complex{Float64}

 [1.0+0.0im 0.0+0.0im 0.0+0.0im;

 0.0+0.0im 0.0+0.0im 0.0+0.0im;

 0.0+0.0im 0.0+0.0im 0.83666+0.0im]

julia> *ρ* = proj(1.0/sqrt(2)*(ket(1,3)+ket(3,3)))

3×3 Array{Complex{Float64},2}:

 0.5+0.0im 0.0+0.0im 0.5+0.0im

 0.0+0.0im 0.0+0.0im 0.0+0.0im

 0.5+0.0im 0.0+0.0im 0.5+0.0im

julia> (PM ∘ Φ)(*ρ*)

3×3 Array{Complex{Float64},2}:

 0.0+0.0im 0.0+0.0im 0.0+0.0im

 0.0+0.0im 0.0+0.0im 0.0+0.0im

 0.0+0.0im 0.0+0.0im 0.35+0.0im

## Random quantum objects

In this section we present the implementation of the sub-package RandomMatrices. The justification for including these functionalities in our package is twofold. First, the application of random matrix theory (RMT) in quantum information is a blooming field of research with a plethora of interesting results [30–39]. Hence, it is useful to have readily available implementations of known algorithms of generating random matrices. Secondly, when performing numerical investigations, we often need “generic” inputs. Generating random matrices with a known distribution is one of the ways to obtain such generic inputs.

### Ginibre matrices

In this section we introduce the Ginibre random matrices ensemble [40]. This ensemble is at the core of a vast majority of algorithms for generating random matrices presented in later subsections. Let (*G*_*ij*_)_1≤*i*≤*m*,1≤*j*≤*n*_ be a *m* × *n* table of independent identically distributed (i.i.d.) random variable on K. The field K can be either of ℝ, ℂ or ℚ. With each of the fields we associate a Dyson index *β* equal to 1, 2, or 4 respectively. Let *G*_*ij*_ be i.i.d random variables with the real and imaginary parts sampled independently from the distribution $N(0,\frac{1}{\beta })$. Hence, G∈L(X,Y),where matrix *G* is

$$P(G)\propto \text{exp}(-\text{Tr}GG^{†}).$$ (7)

This law is unitarily invariant, meaning that for any unitary matrices *U* and *V*, *G* and *UGV* are equally distributed. It can be shown that for *β* = 2 the eigenvalues of *G* are uniformly distributed over the unit disk on the complex plane [41].

In our library the ensemble Ginibre matrices is implemented in the GinibreEnsemble{*β*} parametric type. The parameter determines the Dyson index. The following constructors are provided

julia> GinibreEnsemble{*β*}(m::Int, n::Int)

julia> GinibreEnsemble{*β*}(m::Int)

julia> GinibreEnsemble(m::Int, n::Int)

julia> GinibreEnsemble(m::Int)

The parameters *n* and *m* determine the dimensions of output and input spaces. The versions with one argument assume *m* = *n*. When the Dyson index is omitted it assumed that *β* = 2. Sampling from these distributions can be performed as follows

julia> g = GinibreEnsemble{2}(2,3)

 GinibreEnsemble{2}(m = 2, n = 3)

julia> rand(g)

2×3 Array{Complex{Float64},2}:

 0.835803+1.10758im -0.622744-0.130165im -0.677944+0.636562im

 1.32826+0.106582im -0.460737-0.531975im -0.656758+0.0244259im

The function rand has specialized methods for each possible value of the Dyson index *β*.

### Wishart matrices

Wishart matrices form an ensemble of random positive semidefinite matrices. They are parametrized by two factors. First is the Dyson index *β* which is equal to one for real matrices, two for complex matrices and four for symplectic matrices. The second parameter, *K*, is responsible for the rank of the matrices. They are sampled as follows

1. Choose *β* and *K*.
2. Sample a Ginibre matrix G∈L(X,Y) with the Dyson index *β* and dim(X)=d and dim(Y)=Kd.
3. Return *GG*^†^.

In QuantumInformation.jl this is implemented using the type WishartEnsemble{*β*, K}. We also provide additional constructors for convenience

WishartEnsemble{*β*}(d::Int) where *β* = WishartEnsemble{*β*, 1}(d)

WishartEnsemble(d::Int) = WishartEnsemble{2}(d)

These can be used in the following way

julia> w = WishartEnsemble{1,0.2}(5)

 WishartEnsemble{1,0.2}(d = 5)

julia> z = rand(w)

5×5 Array{Float64,2}:

 0.0897637 0.0257443 0.0314593 0.0223569 0.093517

 0.0257443 0.00738347 0.00902253 0.00641196 0.0268207

 0.0314593 0.00902253 0.0110254 0.00783535 0.0327746

 0.0223569 0.00641196 0.00783535 0.00556828 0.0232917

 0.093517 0.0268207 0.0327746 0.0232917 0.0974271

julia> eigvals(z)

5-element Array{Float64,1}:

 -1.549149323294561e-17

 -1.11670454111383e-18

 1.5797866551971292e-18

 6.408793727745745e-18

 0.21116803949130986

julia> w = WishartEnsemble(3)

 WishartEnsemble{2,1}(d = 3)

julia> z = rand(w)

3×3 Array{Complex{Float64},2}:

 0.474628+0.0im 0.177244-0.0227445im 0.137337-0.0929298im

 0.177244+0.0227445im 0.128676+0.0im 0.0938587-0.165916im

 0.137337+ 0.0929298im 0.0938587+0.165916im 0.555453+0.0im

julia> eigvals(z)

3-element Array{Float64,1}:

 0.01707438064450695

 0.35884924300093163

 0.7828337014291611

### Circular ensembles

Circular ensembles are measures on the space of unitary matrices. There are three main circular ensembles. Each of this ensembles has an associated Dyson index *β* [42]

- Circular orthogonal ensemble (COE), *β* = 1.
- Circular unitary ensemble (CUE), *β* = 2.
- Circular symplectic ensemble (CSE), *β* = 4.

They can be characterized as follows. The CUE is simply the Haar measure on the unitary group. Now, if *U* is an element of CUE then *U*^*T*^ *U* is an element of *COE* and *U*_*R*_ *U* is an element CSE. Here

$$\begin{array}{c} U_{R}=(\begin{array}{ccccccc} 0 & -1 & & & & & \\ 1 & 0 & & & & & \\ & & 0 & -1 & & & \\ & & 1 & 0 & & & \\ & & & & \ddots & & \\ & & & & & 0 & -1\\ & & & & & 1 & 0 \end{array})U^{T}(\begin{array}{ccccccc} 0 & 1 & & & & & \\ -1 & 0 & & & & & \\ & & 0 & 1 & & & \\ & & -1 & 0 & & & \\ & & & & \ddots & & \\ & & & & & 0 & 1\\ & & & & & -1 & 0 \end{array}). \end{array}$$ (8)

As can be seen the sampling of Haar unitaries is at the core of sampling these ensembles. Hence, we will focus on them in the remainder of this section.

There are several possible approaches to generating random unitary matrices according to the Haar measure. One way is to consider known parametrizations of unitary matrices, such as the Euler [43] or Jarlskog [44] ones. Sampling these parameters from appropriate distributions yields a Haar random unitary. The downside is the long computation time, especially for large matrices, as this involves a lot of matrix multiplications. We will not go into this further, we refer the interested reader to the papers on these parametrizations.

Another approach is to consider a Ginibre matrix G∈L(X) and its polar decomposition *G* = *UP*, where U∈L(X) is unitary and *P* is a positive matrix. The matrix *P* is unique and given by $\sqrt{G^{†}G}$. Hence, assuming *P* is invertible, we could recover *U* as

$$\begin{array}{c} U=G{\left(G^{†}G\right)}^{-\frac{1}{2}}. \end{array}$$ (9)

As this involves the inverse square root of a matrix, this approach can be potentially numerically unstable.

The optimal approach is to utilize the QR decomposition of *G*, *G* = *QR*, where Q∈L(X) is unitary and R∈L(X) is upper triangular. This procedure is unique if *G* is invertible and we require the diagonal elements of *R* to be positive. As typical implementations of the QR algorithm do not consider this restriction, we must enforce it ourselves. The algorithm is as follows

1. Generate a Ginibre matrix G∈L(X), dim(X)=d with Dyson index *β* = 2.
2. Perform the QR decomposition obtaining *Q* and *R*.
3. Multiply the *i*^th^ column of *Q* by *r*_*ii*_/|*r*_*ii*_|.

This gives us a Haar distributed random unitary. For detailed analysis of this algorithm see [27]. This procedure can be generalized in order to obtain a random isometry. The only required changed is the dimension of *G*. We simply start with G∈L(X,Y), where dim(X)≥dim(Y).

Furthermore, we may introduce two additional circular ensembles corresponding to the Haar measure on the orthogonal and symplectic groups. These are the circular real ensemble (CRE) and circular quaternion ensemble (CQE). Their sampling is similar to sampling from CUE. The only difference is the initial Dyson index of the Ginibre matrix. This is set to *β* = 1 for CRE and *β* = 4 for CQE.

In QuantumInformation.jl these distributions can be sampled as

julia> c = CircularEnsemble{2}(3)

CircularEnsemble{2}(

d: 3

g: GinibreEnsemble{2}(m = 3, n = 3)

)

julia> u = rand(c)

3×3 Array{Complex{Float64},2}:

 0.339685+0.550434im -0.392266-0.3216im -0.53172+0.203988im

 0.515118-0.422262im 0.392165-0.626859im -0.0504431-0.084009im

 0.297203+0.222832im -0.418737-0.143578im 0.607012-0.545525im

julia> u*u’

3×3 Array{Complex{Float64},2}:

 1.0+0.0im -5.55112e-17-5.55112e-17im -2.77556e-17-4.16334e-17im

 -5.55112e-17+5.55112e-17im 1.0+0.0im -2.498e-16+0.0im

 -2.77556e-17+4.16334e-17im -2.498e-16+0.0im 1.0+0.0im

Sampling from the Haar measure on the orthogonal group can be achieved as

julia> c = CircularRealEnsemble(3)

CircularRealEnsemble(

d: 3

g: GinibreEnsemble{1}(m = 3, n = 3)

)

julia> o = rand(c)

3×3 Array{Float64,2}:

 0.772464 0.611349 -0.171907

 0.0524376 0.208368 0.976644

 0.63289 -0.763436 0.128899

julia> o*o’

3×3 Array{Float64,2}:

 1.0 -1.38778e-16 -8.67362e-17

 -1.38778e-16 1.0 8.32667e-17

 -8.67362e-17 8.32667e-17 1.0

For convenience we provide the following type aliases

const COE = CircularEnsemble{1}

const CUE = CircularEnsemble{2}

const CSE = CircularEnsemble{4}

### Random quantum states

In this section we discuss the properties and methods of generating random quantum states. We will treat quantum channels as a special case of quantum states.

#### Pure states

Pure states are elements of the unit sphere in X. Thus it is straightforward to generate them randomly. We start with a vector of dim(X) independent complex numbers sampled from the standard normal distribution. What remains is to normalize the length of this vector to unity.

This is implemented using the HaarKet{*β*} type. The value *β* = 1 corresponds to the Haar measure on the unit sphere in ℝ^*d*^, while *β* = 2 corresponds to the Haar measure on the unit sphere in ℂ^*d*^. The usage is as follows

julia> h = HaarKet{2}(3)

HaarKet{2}(d = 3)

julia> *ψ* = rand(h)

3-element Array{Complex{Float64},1}:

 0.1687649644765863 − 0.3201009507269653im

 0.7187423269572294 − 0.39405022770434767im

 0.1342475675218075 + 0.42327915636096036im

julia> norm(*ψ*)

 1.0

For convenience we provide the following constructor

HaarKet(d::Int) = HaarKet{2}(d)

as the majority of uses cases require sampling complex states.

#### Mixed states

Random mixed states can be generated in one of two equivalent ways. The first one comes from the partial trace of random pure states. Suppose we have a pure state ψ〉∈X⊗Y. Then we can obtain a random mixed as

$$\begin{array}{c} \rho ={\text{tr}}_{Y}|\psi \rangle \langle \psi |. \end{array}$$ (10)

Note that in the case dim(X)=dim(Y) we recover the (flat) Hilbert-Schmidt distribution on the set of quantum states.

An alternative approach is to start with a Ginibre matrix G∈L(X,Y). We obtain a random quantum state *ρ* as

$$\begin{array}{c} \rho =GG^{†}/\text{Tr}\left(GG^{†}\right). \end{array}$$ (11)

It can be easily verified that this approach is equivalent to the one utilizing random pure states. First, note that in both cases we start with dim(X)dim(Y) complex random numbers sampled from the standard normal distribution. Next, we only need to note that taking the partial trace of a pure state |*ψ*〉 is equivalent to calculating *AA*^†^ where *A* is a matrix obtained from reshaping |*ψ*〉.

The properties of these states have been extensively studied. We will omit stating all the properties here and refer the reader to [31–36].

Sampling random mixed states is implemented using the HilbertSchmidtStates{*β*, K} type. The meaning of the type parameters is the same as in the Wishart matrices case. We provide additional constructors which set the default values of the parameters

HilbertSchmidtStates{*β*}(d::Int) where *β* = HilbertSchmidtStates{*β*, 1}(d)

HilbertSchmidtStates(d::Int) = HilbertSchmidtStates{2, 1}(d)

The latter one is the most frequent use case. Here is an example

julia> h = HilbertSchmidtStates(3)

HilbertSchmidtStates{2,1}(WishartEnsemble{2,1}(d = 3), 3)

julia> *ρ* = rand(h)

3×3 Array{Complex{Float64},2}:

 0.335603+0.0im 0.0696096+0.0606972im 0.0373103+0.0853966im

 0.0696096-0.0606972im 0.209561+0.0im -0.000865656+0.0129982im

 0.0373103-0.0853966im -0.000865656-0.0129982im 0.454836+0.0im

julia> tr(*ρ*)

 1.0 + 0.0im

julia> eigvals(*ρ*)

3-element Array{Float64,1}:

 0.15460054248543945

 0.3306739537037592

 0.5147255038108014

### Random quantum channels

Quantum channels are a special subclass of quantum states with constraints imposed on their *partial* trace as well as trace. Formally, we start with a Ginibre matrix G∈L(X⊗Y,𝒵). We obtain a random Choi-Jamiołkowski matrix *J*_Φ_ corresponding to a channel Φ as

$$\begin{array}{c} J_{\Phi }=(I_{X}⊗{\left({\text{Tr}}_{X}GG^{†}\right)}^{-1/2})GG^{†}(I_{X}⊗{\left({\text{Tr}}_{X}GG^{†}\right)}^{-1/2}). \end{array}$$ (12)

When dim(𝒵)=dim(X)dim(Y) this is known to generate a uniform distribution over the set of quantum channels [37, 38].

The implementation uses the type ChoiJamiolkowskiMatrices{*β*, K}. The parameters *β* and *K* have the same meaning as in the Wishart matrix case. Additionally here, the constructor

ChoiJamiolkowskiMatrices{*β*, K}(idim::Int, odim::Int) where {*β*, K}

takes two parameters—the input and output dimension of the channel. As in the previous cases we provide some additional constructors for convenience

function ChoiJamiolkowskiMatrices{*β*}(idim::Int, odim::Int) where *β*

 ChoiJamiolkowskiMatrices{*β*, 1}(idim, odim)

end

function ChoiJamiolkowskiMatrices{*β*}(d::Int) where *β*

 ChoiJamiolkowskiMatrices{*β*}(d, d)

end

function ChoiJamiolkowskiMatrices(idim::Int, odim::Int)

 ChoiJamiolkowskiMatrices{2}(idim, odim)

end

function ChoiJamiolkowskiMatrices(d::Int)

 ChoiJamiolkowskiMatrices(d, d)

end

Here is an example of usage

julia> c = ChoiJamiolkowskiMatrices(2, 3)

ChoiJamiolkowskiMatrices{2,1}(WishartEnsemble{2,1}(d = 6), 2, 3)

julia> Φ = rand(c)

DynamicalMatrix{Array{Complex{Float64},2}}

dimensions: (2, 3)

Complex{Float64}

 [0.307971-4.98733e-18im -0.00411588+0.0368471im…

 -0.0676732+0.024328im 0.0860858+0.00302876im;

 -0.00411588-0.0368471im 0.167651+2.1684e-19im…

 -0.0428561+0.0266119im 0.0191888+0.0101013im;

 … ;

 -0.0676732-0.024328im -0.0428561 − 0.0266119im…

 0.210419+0.0im -0.103401 − 0.142753im;

 0.0860858-0.00302876im 0.0191888 − 0.0101013im…

 -0.103401+0.142753im 0.411068+0.0im]

julia> ptrace(Φ.matrix, [3, 2],[1])

2×2 Array{Complex{Float64},2}:

 1.0 − 1.53957e-17im  − 1.38778e-17 − 3.05311e-16im

 1.38778e-17 + 3.05311e-16im 1.0 + 2.1684e-19im

Note that the resulting sample is of type DynamicalMatrix.

## Example

As an example we provide the teleportation protocol in the presence of noise. Imagine we have an entangled pair of particles in the state

$$\begin{array}{c} |\psi \rangle =\frac{1}{\sqrt{2}}(|00\rangle +|11\rangle ). \end{array}$$ (13)

One of the particles stays with Alice and another is sent through a noisy channel to Bob. As a noise model we chose the amplitude damping channel given by the Kraus operators

$$\begin{array}{c} K_{0}=(\begin{array}{cc} 1 & 0\\ 0 & \sqrt{1-\gamma } \end{array})\;K_{1}=(\begin{array}{cc} 0 & \sqrt{\gamma }\\ 0 & 0 \end{array}). \end{array}$$ (14)

**Fig 1:**
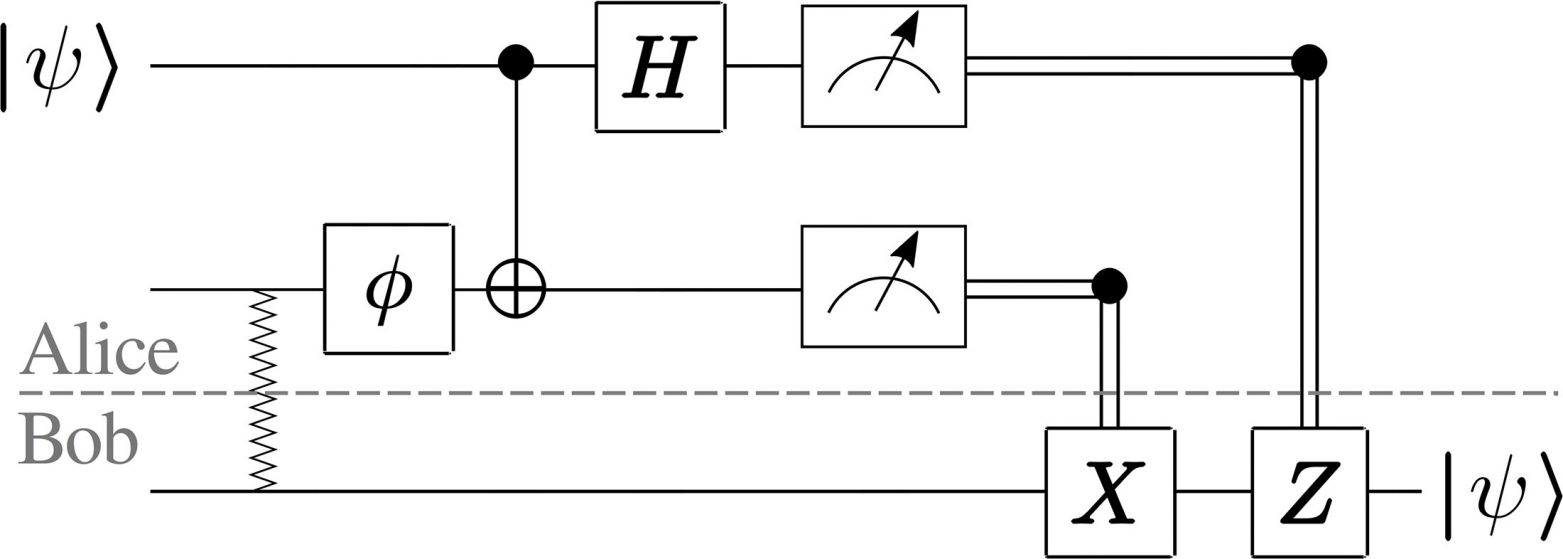
Schematic depiction of the teleportation protocol. Squiggly line represents the maximally entangled state, and box Φ represents the noise operator.

The channel has one parameter *γ* ∈ [0, 1] modeling the strength of the noise. Assume that Alice possesses a random pure state |*ϕ*〉 that she teleports to Bob. The protocol is shown in Fig 1.

Our examples show the fidelity of the final state at Bob’s site averaged over 100 random pure initial states. We also check how the parameter *γ* influences this fidelity.

using QuantumInformation

steps = 100

haar = HaarKet(2)

*ψ* = (ket(0, 4) + ket(3, 4))/sqrt(2)

*γ*s = 0.0:0.01:1.0

Φ = KrausOperators([[1 0; 0 sqrt(1-γ)], [0 sqrt(*γ*); 0 0]])

post = [PostSelectionMeasurement(proj(ket(i, 4)) ⊗ eye(2)) for i = 0:3]

rots = [UnitaryChannel(eye(2)), UnitaryChannel(sx), UnitaryChannel(sz),

UnitaryChannel(sx*sz)]

had = UnitaryChannel{Matrix{ComplexF64}}(hadamard(2))

cnot = UnitaryChannel{Matrix{ComplexF64}}([1 0 0 0; 0 1 0 0; 0 0 0 1; 0 0 1 0])

r = zeros(steps, length(*γ*s), 4);

for (k, *γ*) in enumerate(*γ*s)

 for i = 1:steps

  *ϕ* = rand(haar)

  *ξ* = *ϕ* ⊗ *ψ*

  *ρ* = ((had ⊗ IdentityChannel(4))∘(cnot ⊗

IdentityChannel(2))∘(IdentityChannel(4) ⊗ Φ))(*ξ*)

  for j = 1:4

   *σ* = rots[j](ptrace(post[j](*ρ*), [2, 2, 2], [1, 2]))

   r[i, k, j] = fidelity(*ϕ*, *σ*/tr(*σ*))

  end

 end

end

mean(r, 1)

## Benchmarks

In the benchmarks we compare our library to the state-of-the-art Python library, QuTiP [12, 13]. We perform the following tests:

1. sampling a random unitary matrix,
2. sampling a random pure state,
3. sampling a random mixed state,
4. sampling a random channel,
5. calculating the trace distance of a random mixed state from the maximally mixed state,
6. calculating the trace distance between two random mixed states,
7. calculating the entropy of the stationary state of a random channel.

The latter is done as follows. First we sample a random quantum channel. Next, we apply the reshuffle operation and calculate its eigenvectors and eigenvalues. We take the state corresponding to the eigenvalue equal to one and calculate its von Neumann entropy. All tests are performed 1000 times and an average time of computation from these samples is calculated. In the case of Julia code we ensure all functions are compiled prior to testing. The tests are performed for dimensions 4, 16, 64, 256, 1024. In the next subsections we present and discuss results for all the aforementioned cases.

The tests were performed on a machine equipped with Intel Core i7-6800K and 64 GB of RAM. The libraries installed on the system were

julia> versioninfo()

julia Version 1.0.0

Platform Info:

 OS: Linux (x86_64-redhat-linux)

 CPU: Intel(R) Core(TM) i7-6800K CPU @ 3.40GHz

 WORD_SIZE: 64

 LIBM: libopenlibm

 LLVM: libLLVM − 6.0.0 (ORCJIT, broadwell)

julia> LAPACK.version()

v“3.7.0”

julia> BLAS.vendor()

:openblas

julia> BLAS.openblas_get_config()

“DYNAMIC_ARCH_NO_AFFINITY Haswell”

The Python libraries were

In [1]: import numpy as np

In [2]: np.__version__

Out[2]: ‘1.7.1’

In [3]: np.__config__.show()

Out[3]:

blas_mkl_info:

 NOT AVAILABLE

blis_info:

 NOT AVAILABLE

openblas_info:

 libraries = [‘openblas’, ‘openblas’]

 library_dirs = [‘/home/user/anaconda3/lib’]

 language = c

 define_macros = [(‘HAVE CBLAS’, None)]

blas_opt_info:

 libraries = [‘openblas’, ‘openblas’]

 library_dirs = [‘/home/user/anaconda3/lib’]

 language = c

 define_macros = [(‘HAVE CBLAS’, None)]

lapack_mkl_info:

 NOT AVAILABLE

openblas_lapack_info:

 libraries = [‘openblas’, ‘openblas’]

 library_dirs = [‘/home/user/anaconda3/lib’]

 language = c

 define_macros = [(‘HAVE CBLAS’, None)]

lapack_opt_info:

 libraries = [‘openblas’, ‘openblas’]

 library_dirs = [‘/home/user/anaconda3/lib’]

 language = c

 define_macros = [(‘HAVE CBLAS’, None)]

### Sampling a random unitary matrix

The Julia code for this test is

using QuantumInformation

function random_unitary(steps::Int, d::Int)

 dist = CUE(d)

 for i = 1:steps U = rand(dist) end

end

The Python implementation reads

import qutip as q

def random_unitary(steps, d):

 for _ in range(steps):

  q.rand_unitary_haar(d)

**Fig 2:**
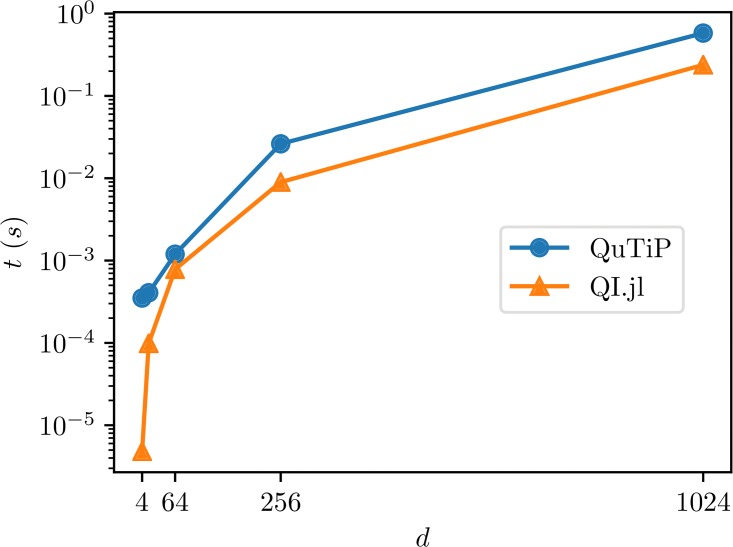
Benchmark results for sampling random unitary matrices in QuantumInformation.jl and Python.

The benchmark results are presented in Fig 2. Note that, as advertised, our implementation is faster and the gap gets bigger as the dimension of the input system increases.

### Sampling a random pure state

The Julia code for this test is

using QuantumInformation

function random_pure_state(steps::Int, d::Int)

 dist = HaarKet(d)

 for i = 1:steps *ψ* = rand(dist) end

end

The Python implementation reads

import qutip as q

def random_pure_state(steps, d):

 for _ in range(steps):

  q.rand_ket_haar(d)

**Fig 3:**
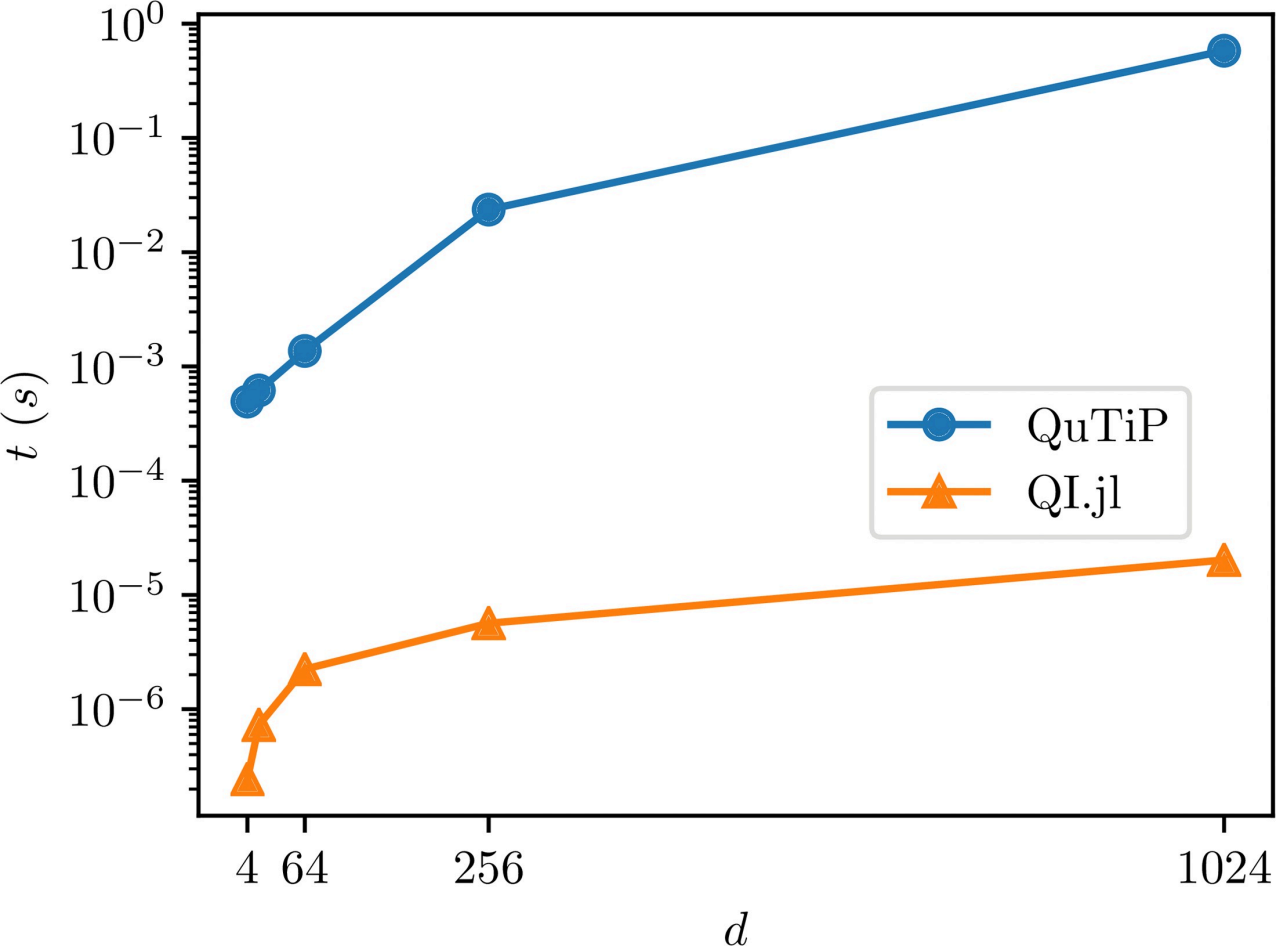
Benchmark results for sampling random pure states in QuantumInformation.jl and Python.

The benchmark results are presented in Fig 3. In this case we get a huge difference in the computation times. This is due to the fact that QuTiP first samples an entire random unitary matrix and returns its first column as the sampled state. On the other hand our implementation samples only one vector.

### Sampling a random mixed state

The Julia code for this test is

using QuantumInformation

function random_mixed_state(steps::Int, d::Int)

 dist = HilbertSchmidtStates(d)

 for i = 1:steps *ρ* = rand(dist) end

end

The Python implementation reads

import qutip as q

def random_mixed_state(steps, d):

 for _ in range(steps):

  q.rand_dm_hs(d)

**Fig 4:**
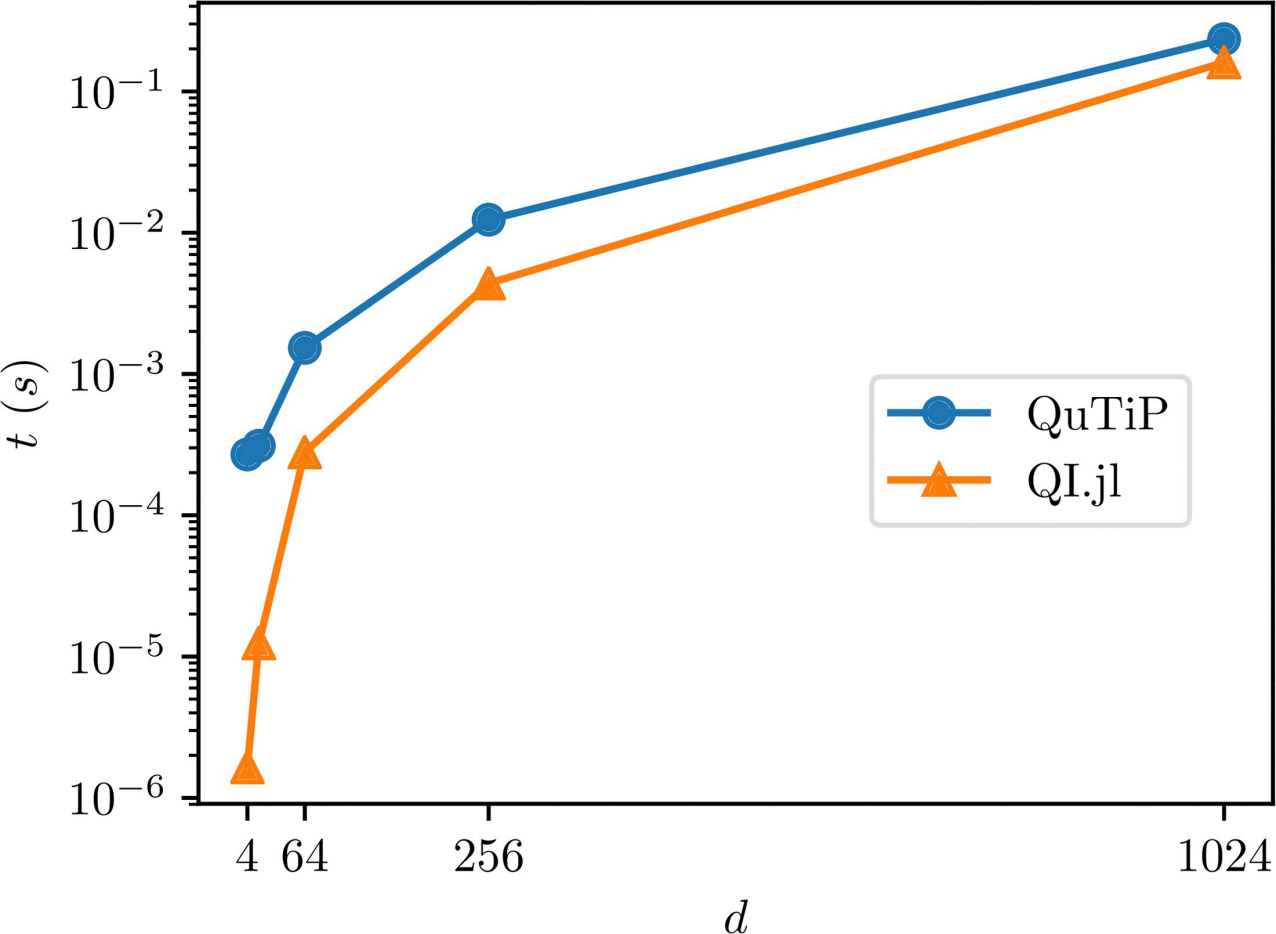
Benchmark results for sampling random mixed state in QuantumInformation.jl and Python.

The benchmark results are presented in Fig 4. Again, our package is faster compared to QuTiP.

### Sampling a random channel

The Julia code for this test is

using QuantumInformation

function random_channel(steps::Int, d::Int)

 dist = ChoiJamiolkowskiMatrices(round(Int, sqrt(d)))

 for i = 1:steps Φ = convert(SuperOperator{Matrix{ComplexF64}}, rand(dist)) end

end

The Python implementation reads

import qutip as q

def random_channel(steps, d):

 for _ in range(steps):

  q.rand_super_bcsz(int(np.sqrt(d)))

**Fig 5:**
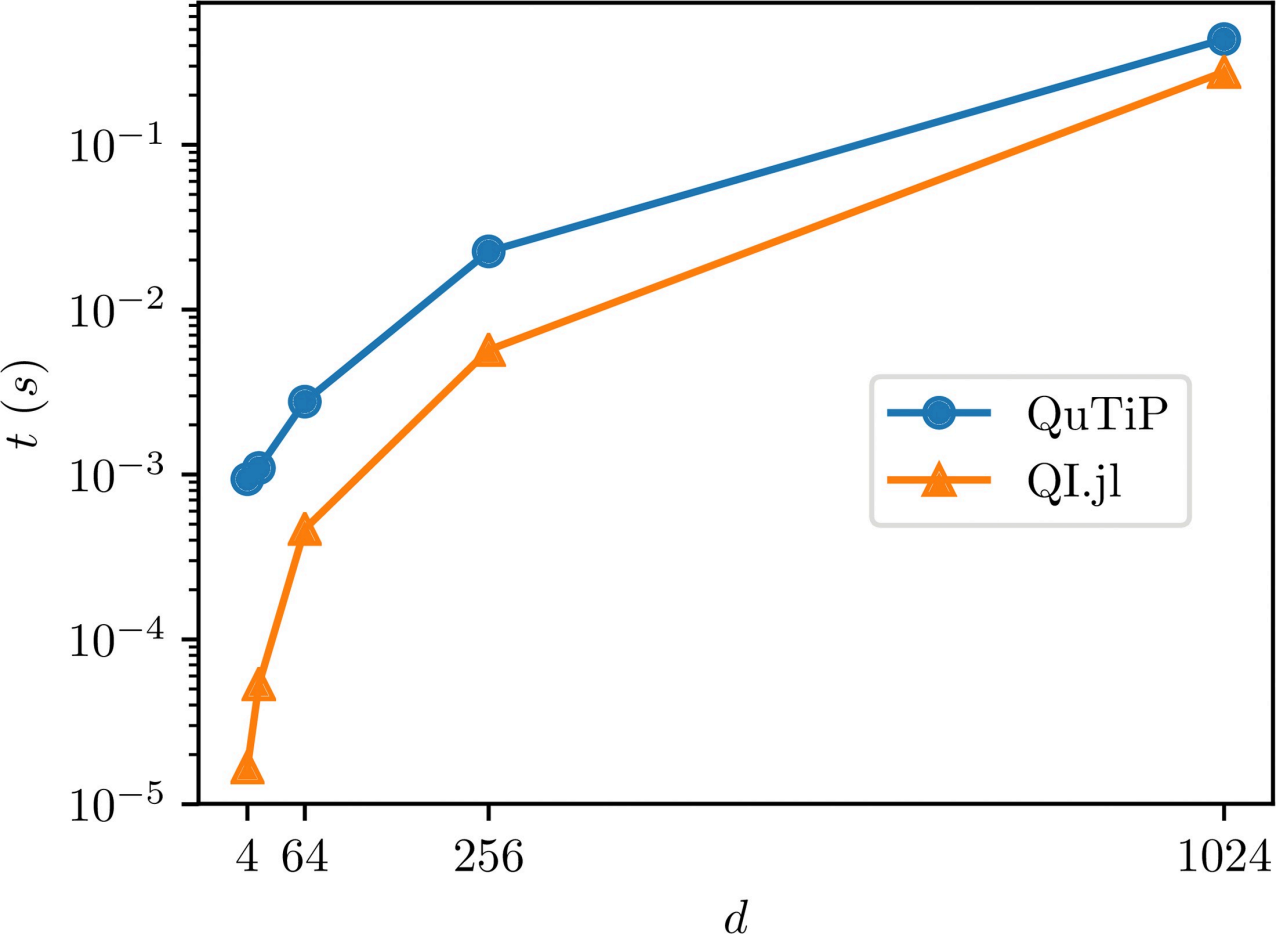
Benchmark results for sampling random quantum channels in QuantumInformation.jl and Python.

Note the conversion to SuperOperator in the benchmark. This is to mimic QuTiP’s behavior which returns a superoperator. The benchmark results are presented in Fig 5.

### Calculating the trace distance form the maximally mixed state

The Julia code for this test is

using QuantumInformation

function trace_distance_max_mixed(steps::Int, d::Int)

 dist = HilbertSchmidtStates(d)

 ρ=I(d)/d

 for i = 1:steps trace distance(rand(dist), *ρ*) end

end

The Python implementation reads

import qutip as q

def trace_distance_max_mixed(steps, d):

 rho = q.Qobj(np.eye(d) / d)

 for _ in range(steps):

  q.metrics.tracedist(q.rand_dm_hs(d), rho)

**Fig 6:**
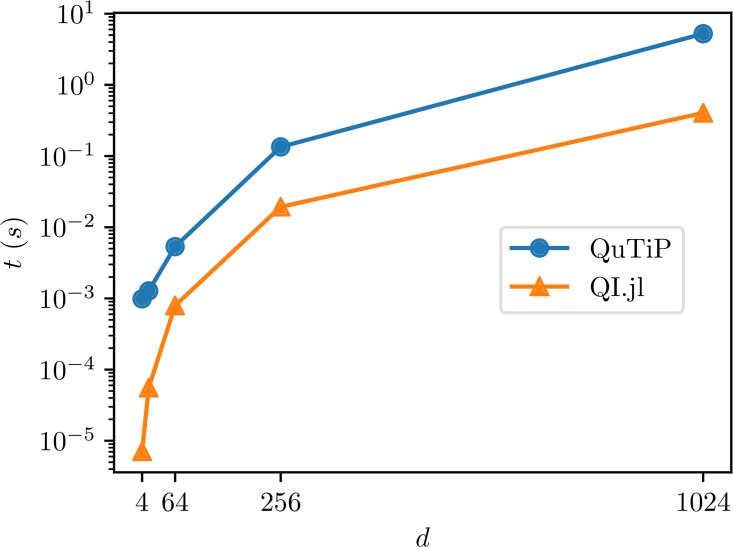
Benchmark results for calculating the trace distance between a random mixed state and the maximally mixed in QuantumInformation.jl and Python.

The benchmark results are presented in Fig 6. Again, for all studied dimensions, our implementation is faster compared to Python.

### Calculating the trace distance between two random mixed states

The Julia code for this test is

using QuantumInformation

function trace_distance_random(steps::Int, d::Int)

 dist = HilbertSchmidtStates(d)

 for i = 1:steps trace_distance(rand(dist), rand(dist)) end

end

The Python implementation reads

import qutip as q

def trace_distance_random(steps, d):

 for _ in range(steps):

  q.metrics.tracedist(q.rand_dm_hs(d), q.rand_dm_hs(d))

**Fig 7:**
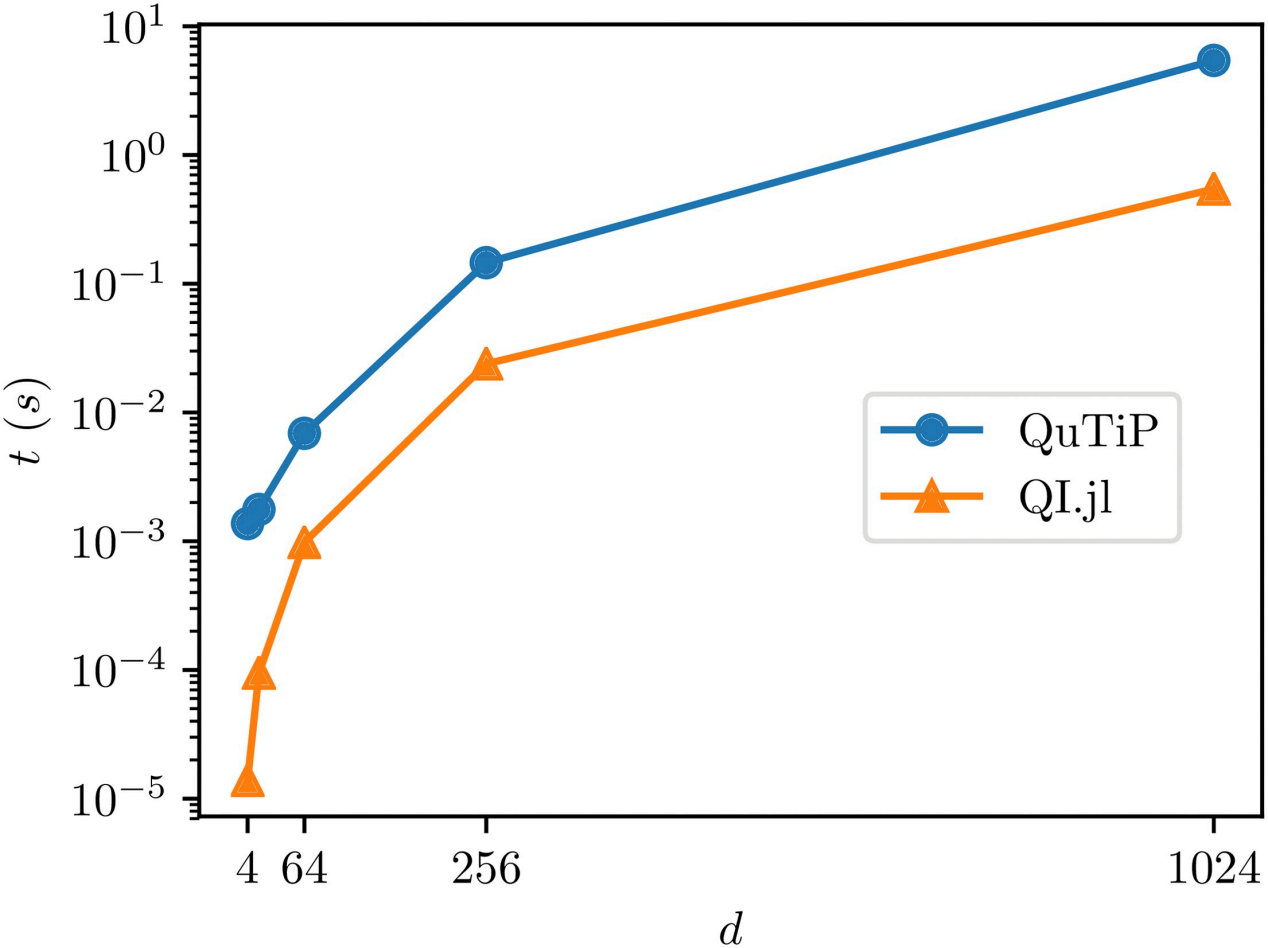
Benchmark results for calculating the trace distance between two random mixed states in QuantumInformation.jl and Python.

The benchmark results are presented in Fig 7.

### Calculating the entropy of the stationary state of a random channel

The Julia code for this test is

using QuantumInformation

function random_unitary(steps::Int, d::Int)

 dist = CUE(d)

 for i = 1:steps U = rand(dist) end

end

The Python implementation reads

import qutip as q

def random_unitary(steps, d):

 for _ in range(steps):

  q.rand_unitary_haar(d)

**Fig 8:**
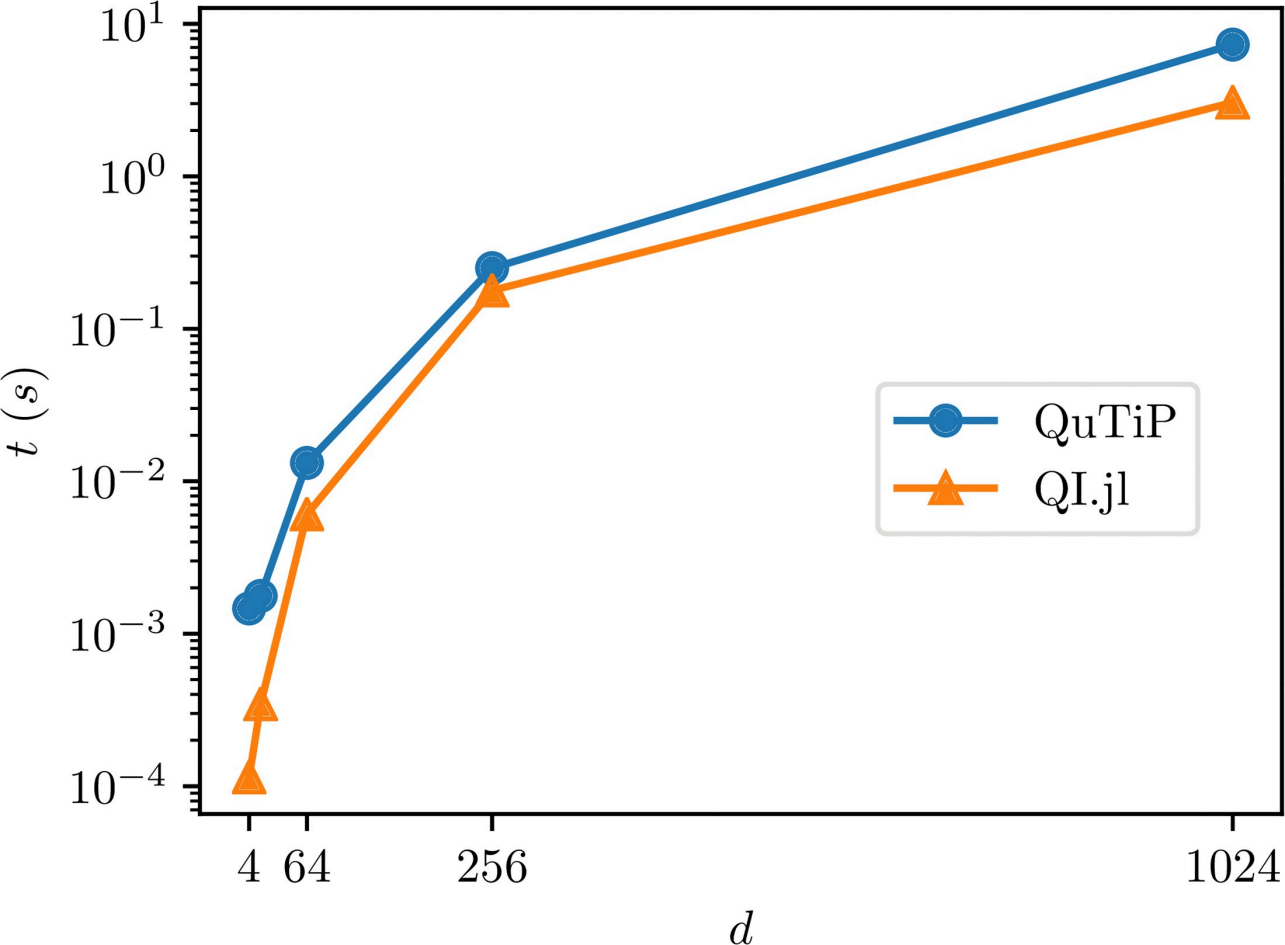
Benchmark results for calculating the entropy of a stationary state of a random quantum channel in QuantumInformation.jl and Python.

The benchmark results are presented in Fig 8.

## Conclusions and future work

Numerical investigations are important part of research in many fields of science, especially in quantum information. The Julia language is a modern programming language, which provides strong support for linear algebra and posses an extensive type system. One of the important feature of Julia is high performance approaching statically-compiled languages like C or Fortran. Those were the reasons why we created the QuantumInformation.jl library in Julia.

We performed benchmark comparisons of QuantumInformation.jl with QuTiP. They clearly state that our library is faster compared to the current state of the art. As the core numerical libraries were the same for both tested packages, we conclude that this speedup is due to the advantages offered by Julia.

Future work will consists of optimization of numerical code, extending the type system, developing further functionals, better integration with Convex.jl package. Additional work will also include parallelization of the code and support for writing quantum circuits in more intuitive manner.
